# Vital-sign circadian rhythms in patients prior to discharge from an ICU: a retrospective observational analysis of routinely recorded physiological data

**DOI:** 10.1186/s13054-020-02861-2

**Published:** 2020-04-28

**Authors:** Shaun Davidson, Mauricio Villarroel, Mirae Harford, Eoin Finnegan, Joao Jorge, Duncan Young, Peter Watkinson, Lionel Tarassenko

**Affiliations:** 1 Institute of Biomedical Engineering, Department of Engineering Science, University of Oxford, Oxford, UK; 2 Critical Care Research Group, Nuffield Department of Clinical Neurosciences, University of Oxford, Oxford, UK

**Keywords:** Circadian rhythms, Intensive care unit, Blood pressure, Vital-sign monitoring, Delirium

## Introduction

Patient care in an intensive care unit (ICU) typically involves maintaining homeostasis or ‘normalisation’ of vital signs [1–3], where the body is unable to provide this for itself. However, the process of controlling and regulating vital signs, combined with sedation, inflammation, environmental light, and noise levels, can disrupt a patient’s natural circadian rhythms [4]. ICU practice in general does not emphasise support of a patient’s circadian rhythms, though there is a growing desire to improve upon this [5]. Chronically disrupted circadian rhythms are associated with metabolic disorders such as obesity and diabetes, cardiovascular disease, and cancer [6–9]. In an ICU, disruption or loss of a patient’s circadian rhythms is associated with complications such as immune system disruption [10], delirium [11, 12], and mortality [13, 14].

The assessment of circadian behaviour in the ICU typically focuses on the study of sleep, ideally recorded using polysomnography [15, 16]. However, difficulties with instrumentation in the ICU [17], abnormal electroencephalography (EEG, brain activity) patterns [16, 18, 19], and the relative sensitivity of sleep to events such as lighting or environmental noise variations mean that sleep is not necessarily an ideal or easily established marker of patient circadian behaviour. Thus, a recent review commented that ‘Finding the optimum tool to monitor (circadian rhythms in) critically ill patients therefore remains a key to research progress in this area’ [3]. Healthy individuals exhibit circadian rhythms in several vital signs, including systolic blood pressure (SBP), heart rate (HR), respiratory rate (RR), and core body temperature (T) [3]. However, the ‘severe circadian deregulation’ [4] experienced by patients treated in ICUs can result in abnormal vital-sign patterns.

Several studies have established typical circadian vital-sign behaviour in healthy individuals. Hermida et al. [20] conducted a study using ambulatory monitoring on 278 healthy individuals with mean ± SD age 22.7 ± 3.3 years, synchronising measurements to individual sleep/wake times rather than time of day. They observed an elevated SBP during the day, which reached an approximate plateau (with 3 periodic local maxima) between ≈ 3 and ≈ 13 h after awakening, followed by a sinusoidal dip during sleep. The observed rhythms in HR closely corresponded to those observed in SBP, with an elevated plateau (again with 3 periodic local maxima) between ≈ 2 and ≈ 14 h after awakening, decreasing slightly during the day, and a sinusoidal dip overnight.

Bosco et al. [21] conducted a study in which 6 males (competitive scuba divers with mean ± SEM age 39 ± 3 years) were kept in a constant routine protocol (sustained wakefulness, minimal activity). RR was observed to peak late in the day (≈ 8 pm) with a trough at ≈ 3–7 am, roughly in phase with HR. Spengler et al. [22] conducted a similar study in which 10 healthy males with mean ± SD age 23.7 ± 3.9 years were kept in a relaxed, semi-recumbent position isolated from any indication of time of day for 41 h. As in [20], measurements were synchronised to individual sleep/wake times. While they do not report RR, Spengler et al. report ventilation (V _E_) in l/min, which was elevated between ≈ 2 h before awakening and ≈ 8 h after awakening, and decreased to an approximate plateau overnight. Core body temperature showed an approximately sinusoidal form, with nadir that lagged behind the nadir in V _E_ by ≈ 6–8 h.

Given previous work has suggested circadian behaviour is severely disrupted in an ICU [3, 4, 15, 23], and assuming that circadian behaviour in the majority of patients returns to normality post-ICU discharge, patients treated in an ICU undergo a ‘circadian recovery’ process as part of their overall recovery. If this ‘circadian recovery’ process was shown to begin prior to ICU discharge in patients who subsequently recovered (i.e. were discharged home), and this circadian state was shown to be generalisable across different ICU populations (i.e. not due to external behaviour such as nursing shift changes), there are several potential clinical applications. These include the monitoring of ICU patient recovery, as well as monitoring the development of complications associated with disrupted circadian rhythms such as delirium. We hypothesise that vital-sign circadian rhythms will be observable in the 24 h prior to discharge from the ICU in patients who subsequently fully recovered, that these circadian rhythms will resemble known behaviour in healthy individuals, and that these circadian rhythms will be generalisable across different populations of ICU patients. We set out to validate these hypotheses across three large, retrospective clinical databases.

## Materials and methods

### Databases

This study makes use of three clinical databases:

- Medical Information Mart for Intensive Care III (MIMIC-III) is a database of critical care information gathered at the Beth Israel Deaconess Medical Centre (BIDMC) in Boston, MA, USA, between 2001 and 2012 [24, 25].
- The eICU Collaborative Research Database (eICU-CRD) is a database of critical care information gathered from 208 hospitals across the continental USA between 2014 and 2015 [26].
- The Post-Intensive-Care Risk-adjusted Alerting and Monitoring (PICRAM) database (ISRCTN32008295) includes patients admitted to the adult ICU or coronary care unit at the John Radcliffe Hospital, Oxford, UK, between 2008 and 2015, as well as patients admitted to the ICU at the Reading Berkshire Hospital, Reading, UK, between 2009 and 2015.

Access to the MIMIC-III and eICU-CRD databases for the purpose of this study was granted by the institutional review boards of the BIDMC and Massachusetts Institute of Technology (Cambridge, MA, USA). Access to the PICRAM database was granted by the Critical Care Research Group Data Access Committee of the University of Oxford. Combined, these databases span 211 hospitals across two countries, with different patient demographics, standards of clinical practice, and use of equipment.

### Data selection

Data from the MIMIC-III, eICU-CRD, and PICRAM databases were selected according to the following criteria:

1. The patient must have had at least one cuff SBP reading recorded.
2. The patient must not have died over the course of the given hospital stay, nor have been discharged to hospice (end of life) care.
3. In the case of MIMIC-III and eICU-CRD, the patient must have been discharged ‘home’ or to ‘home health care’. In the case of PICRAM, the patient must have been discharged with an expected dependency of ‘Able to live without assistance in daily activities’.
4. The patient must not have had any Do Not Resuscitate (DNR), Do Not Intubate (DNI), or ‘Comfort Measures Only’ codes, as this indicates a deviation from typical ICU care.
5. The patient must have been at least 15 years of age.
6. The patient must have spent at least 24 h in an ICU continuously.
7. Only measurements taken during the final 24 h before a patient was discharged from an ICU were included. That is, if a patient was discharged at 11 am, measurements from 11 am on the previous day were included.
8. Measurements outside of the broad physiological bounds (60 mmHg < SBP < 280 mmHg, 30 bpm < HR < 240 bpm, 4 breaths/min < RR < 60 breaths/min, 34^∘^C < T < 40^∘^C) were excluded.
9. Measurements taken while the patient was under the effect of treatments that were likely to significantly affect the vital signs being measured were excluded. This process focused on removing measurements taken while vasopressors, *β*-blockers, or other blood pressure medication were likely to be active, and is discussed in more detail in Additional file 1. Vital-sign measurements were excluded:
  Up to 1 h after a patient was administered dobutamine, dopamine, adrenaline/epinephrine, noradrenaline/norepinephrine, metaraminol, glyceryl trinitrate, dopexamine, nitroprusside, or isoprenaline [27, 28].
  Up to 2 h after a patient was administered vasopressin, propofol, magnesium sulphate, ephedrine, or phentolamine [29–31].
  Up to 24 h after a patient was administered milrinone, terlipressin, labetalol, metoprolol, or hydralazine [27, 32, 33].
10. The patient must have had at least one night-time (12 midnight–5:59 am) and one day-time (10 am–7:59 pm) SBP measurement as in [34]. The majority of patients will have more available SBP measurements than this (see Additional file 2), but this requirement ensures each patient contributes to both ‘day-time’ and ‘night-time’ behaviour.
11. For MIMIC-III and PICRAM, if the patient had multiple ICU stays within 6 months of each other, all ICU stays within this period were excluded due to it being unlikely the patient was discharged ‘healthy’. An ICU stay is defined as a period during which a patient occupied a bed in the ICU, including short-term removal for surgery, scans, or other interventions. A hospital admission is defined as the time between patient admission to and discharge from the hospital. In the eICU-CRD, no relative dates are recorded for hospital admissions. Instead, any hospital admission containing multiple ICU admissions was discarded entirely.
12. For each ICU stay, all measurements in each 1-h period were averaged for each vital sign for the final 24 h of that ICU stay. This process avoids weighting data towards ICU stays where patients are more ill, and thus likely to have more regular vital-sign measurements. These mean hourly values were recorded left aligned (e.g. the mean of measurements between 1:00 am and 1:59 am was recorded as occurring at 1:00 am). Vital signs were typically measured at least hourly, with the exception of temperature in MIMIC-III and PICRAM which was typically measured once every 4 h. If there were no measurements of a given vital sign in a given 1-h period in an ICU stay, that ICU stay did not contribute any measurement for that hour to the overall analysis.

### Data analysis

Patients were separated into groups by gender and age, as there are established trends in mean SBP and HR associated with gender and age [34]. Observation of similar trends in the selected ICU cohorts would give support to the notion that underlying physiological, rather than treatment or pathology driven, behaviour is being observed in these patients. The age groups used in this paper are a modified set of those specified in ‘Provisional guidelines on standard international age classifications’ for ‘health, health services and nutrition - morbidity and handicaps’ [35]. These age groups are as follows: 15–45 years (combining the recommended 15–25- and 25–45-year groups due to the low number of patients under 45 treated in ICUs), 45–65 years, and 65+ years. Per HIPAA regulations, the ages of individuals greater than 89 were not recorded in MIMIC-III or eICU-CRD. These patients were treated as 91 years of age; thus, all fell within the 65+-year age group.

The median Oxford Acute Severity of Illness Score (OASIS), a severity of illness score used for predicting patient outcomes [36], was determined for each patient subgroup. The OASIS was designed to require a minimal set of physiological parameters. In contrast, common severity of illness scores such as APACHE and SAPS employ a wide variety of physiological measurements that are not necessarily well recorded or easy to recover from large ICU databases. As such, OASIS is more easily and consistently applicable across a range of retrospective clinical databases with different recording standards.

Evaluation of circadian rhythmicity was performed using several approaches, which were performed using the 24-h mean vital-sign profiles established for each patient cohort. The observed profiles were visually compared to circadian vital-sign profiles found in the literature, typically available for non-ICU cohorts. As a quantitative indication of rhythm amplitude or strength, the peak-nadir excursion [37] was calculated, expressed as both a raw value and as a percentage of the 24-h mean for that vital-sign profile. These values were compared to values reported in the literature. To evaluate the consistency of corresponding vital-sign profiles between databases, the cross correlation (*R*) and accompanying *p* values (*p*) were calculated. For the correlation analysis, temperature profiles from eICU-CRD were subsampled at 4-hourly intervals to allow for comparison with MIMIC-III and PICRAM.

To provide an indication of intra-cohort variability, the hourly 95% confidence intervals (CIs) of the vital-sign mean were calculated for each 24-h vital-sign profile [38]. Graphically, if two vital-sign CIs do not overlap for any given hour, their means are significantly different at the *p* = 0.05 level. Where comparison between databases is desired, a two-sample Student’s *t* test was used to compare each hourly bin of vital-sign measurements. As before, mean vital-sign levels were deemed significantly different if each of the 24 hourly bins was found to be significantly different at the *p* = 0.05 level.

## Results

### Database and cohort demographics

Tables 1 and 2 present demographic data for the entire databases and the selected cohort from each database, respectively. The overall median age of patients in the selected cohort for PICRAM (61.2 years) was greater than that for the corresponding cohort in MIMIC-III (59.6 years) or eICU-CRD (60.0 years). Similarly, the overall median OASIS of patients in the selected cohort PICRAM (33) was greater than that for MIMIC-III (27) or eICU-CRD (26). These results suggest that on average, the selected PICRAM patients were older and more ill, corresponding to the increased LOS observed in the selected PICRAM cohort (Table 2).

**Table 1:** Overall demographics for each database (no exclusion criteria applied), grouped by gender

	MIMIC-III		eICU-CRD		PICRAM	
Statistic	Men	Women	Men	Women	Men	Women
No. of patients	26,121	20,399	75,188	64,044	7196	5090
No. of hospital admissions	32,950	26,026	89,391	76,802	7810	5559
No. of ICU stays	34,469	27,063	108,379	92,303	8176	5767
Age (years), median (IQR)	61 (30)	64 (34)	64 (22)	66 (25)	65 (24)	62 (27)
ICU LOS^*^ (days), median (IQR)	2.1 (3.3)	2.1 (3.4)	1.6 (2.1)	1.6 (2.2)	2.0 (3.7)	2.0 (3.1)
OASIS, median (IQR)	29 (12)	30 (13)	27 (14)	29 (14)	33 (17)	33 (18)
ICU mortality (%)	7.4	8.0	8.9	9.1	12.8	11.7

^*^Length of stay

**Table 2:** Demographics of the selected cohort of ICU stays from each database, grouped by gender

	MIMIC-III		eICU-CRD		PICRAM	
Age group	Men	Women	Men	Women	Men	Women
Breakdown of no. (%) of ICU stays in the selected cohort by age group						
15–44	1395 (11.8)	1095 (9.2)	3670 (10.4)	3574 (10.2)	412 (12.5)	390 (11.9)
45–64	3054 (25.7)	1869 (15.7)	7991 (22.7)	6089 (17.3)	621 (18.9)	486 (14.8)
65+	2608 (22.0)	1851 (15.6)	7572 (21.5)	6238 (17.8)	844 (25.7)	528 (16.1)
Total	7057 (59.4)	4815 (40.6)	19,233 (54.7)	15,901 (45.2)	1877 (57.2)	1404 (42.8)
Median (IQR) ICU LOS^*^(days)						
15–44	2.1 (2.1)	2.1 (1.8)	1.9 (1.6)	1.8 (1.5)	3.6 (5.2)	3.2 (5.2)
45–64	2.1 (1.8)	2.1 (1.7)	2.0 (1.7)	2.0 (1.6)	3.8 (6.8)	3.3 (4.8)
65+	2.1 (1.7)	2.0 (1.6)	1.9 (1.6)	1.9 (1.5)	3.1 (4.2)	3.7 (4.5)
Overall	2.1 (1.8)	2.1 (1.7)	1.9 (1.6)	1.9 (1.5)	3.4 (5.0)	3.5 (4.7)
Median (IQR) OASIS						
15–44	25 (11)	25 (10)	24 (11)	24 (12)	33 (15)	30 (16)
45–64	26 (9)	27 (10)	25 (12)	26 (12)	33 (16)	33 (17)
65+	28 (9)	29 (9)	27 (11)	29 (11)	33 (18)	34 (18)
Overall	27 (10)	27 (10)	26 (12)	27 (12)	33 (17)	33 (16)

^*^Length of stay

Additionally, median OASIS were identical for the PICRAM cohort selected in this paper and the overall PICRAM database (33), unlike MIMIC-III (27 selected and 29 overall) and eICU-CRD (26 selected and 28 overall). This suggests the employed selection criteria were less discriminatory in PICRAM. This notion is supported by Table 3, which shows the number of patients (#Pat.), hospital admissions (#Hosp.), ICU stays (#ICU), and vital-sign measurements (#SBP, #HR, #RR, #T) that met the cumulative application of the criteria set out previously for each database. In this table, it can be observed that a higher portion of PICRAM ICU stays were retained (23.5%) by the selection process than for MIMIC-III (19.3%) or eICU-CRD (17.5%). Despite this higher retention rate, the selected cohort of PICRAM ICU stays (3283 ICU stays, Table 2) is still significantly smaller than the size of the selected MIMIC-III (11,872 ICU stays) or eICU-CRD (35,143 ICU stays) cohorts.

**Table 3:** The number of patients, hospital admissions, ICU stays, and vital-sign measurements in each database that matched each selection criterion applied cumulatively

Subset	#Patients	#Hosp.	#ICU	#SBP	#HR	#RR	#T
Breakdown of MIMIC-III measurements							
All	46,476	57,786	61,532	2,871,980	7,936,326	6,520,159	1,128,747
1. Cuff SBP	37,671	48,033	51,392	2,871,980	6,168,906	6,433,882	1,114,487
2. Survived	33,483	42,604	45,217	2,455,896	4,991,389	5,158,016	915,228
3. Disch. home	20,376	24,308	25,169	986,392	1,774,032	1,778,552	322,043
4. No DNR/DNI	20,212	24,039	24,888	970,349	1,736,234	1,740,712	318,767
5. Age 15+	20,212	24,039	24,888	970,349	1,736,234	1,740,712	318,767
6. Stay 24 h+	17,017	19,915	20,531	901,239	1,635,417	1,641,151	303,039
7. Last 24 h	16,994	19,883	20,496	320,982	488,386	481,243	87,358
8. Valid meas.	16,994	19,883	20,496	320,560	488,276	480,119	86,769
9. BP medication	16,093	18,760	19,284	281,334	403,196	395,149	69,742
10. Day/night BP	11,886	13,791	14,082	259,502	318,404	311,561	52,165
11. No repeat stays	11,194	11,872	11,872	218,625	266,983	261,084	44,022
12. Hourly avr.	11,194	11,872	11,872	209,595	230,648	225,262	31,628
Breakdown of eICU-CRD measurements							
All	139,367	166,355	200,859	22,079,437	146,070,343	128,586,418	13,267,119
1. Cuff SBP	127,486	151,397	176,497	20,666,164	135,849,528	119,354,199	12,444,242
2. Survived	116,598	138,226	160,825	17,370,839	117,960,824	103,466,212	9,673,554
3. Disch. home	78,489	89,120	101,953	8,119,745	57,392,933	49,471,570	3,637,550
4. No DNR/DNI	74,679	84,478	96,452	7,460,215	53,032,738	45,605,571	3,387,661
5. Age 15+	74,603	84,391	96,362	7,458,391	53,013,037	45,587,254	3,387,661
6. Stay 24 h+	52,904	58,460	62,120	6,567,760	47,237,116	40,566,523	3,227,193
7. Last 24 h	52,655	58,161	61,748	1,946,890	16,122,421	13,714,491	579,237
8. Valid meas.	52,654	58,159	61,746	1,944,573	16,119,746	13,626,000	576,511
9. BP medication	42,269	46,350	48,813	1,474,812	11,904,784	10,183,343	461,021
10. Day/night BP	37,615	41,170	43,086	1,435,798	11,212,410	9,645,215	414,644
11. No repeat stays	32,385	35,143	35,143	1,177,751	9,151,423	7,905,285	346,133
12. Hourly avr.	32,385	35,143	35,143	685,626	790,718	691,886	30,080
Breakdown of PICRAM measurements							
All	12,290	13,138	13,949	334,120	1,295,070	1,306,271	346,474
1. Cuff SBP	11,351	12,113	12,845	334,120	1,265,862	1,277,367	341,646
2. Survived	10,034	10,736	11,382	291,376	1,033,072	1,042,400	287,620
3. Disch. home	7823	8249	8724	195,253	716,926	717,929	200,002
4. No DNR/DNI	7731	8149	8613	188,800	694,441	695,583	195,635
5. Age 15+	7713	8131	8595	188,452	693,344	694,510	195,063
6. Stay 24 h+	5971	6268	6608	178,371	663,006	664,570	185,809
7. Last 24 h	5970	6267	6607	43,195	100,657	99,859	35,688
8. Valid meas.	5970	6267	6607	43,049	100,521	99,786	35,562
9. BP medication	5877	6156	6488	40,464	88,717	88,232	32,782
10. Day/night BP	3385	3480	3613	33,199	44,298	44,154	18,441
11. No repeat stays	3237	3282	3283	30,090	40,213	40,091	16,768
12. Hourly avr.	3237	3282	3283	29,572	39,628	39,520	16,694

### Circadian vital-sign qualitative analyses

**Fig. 1:**
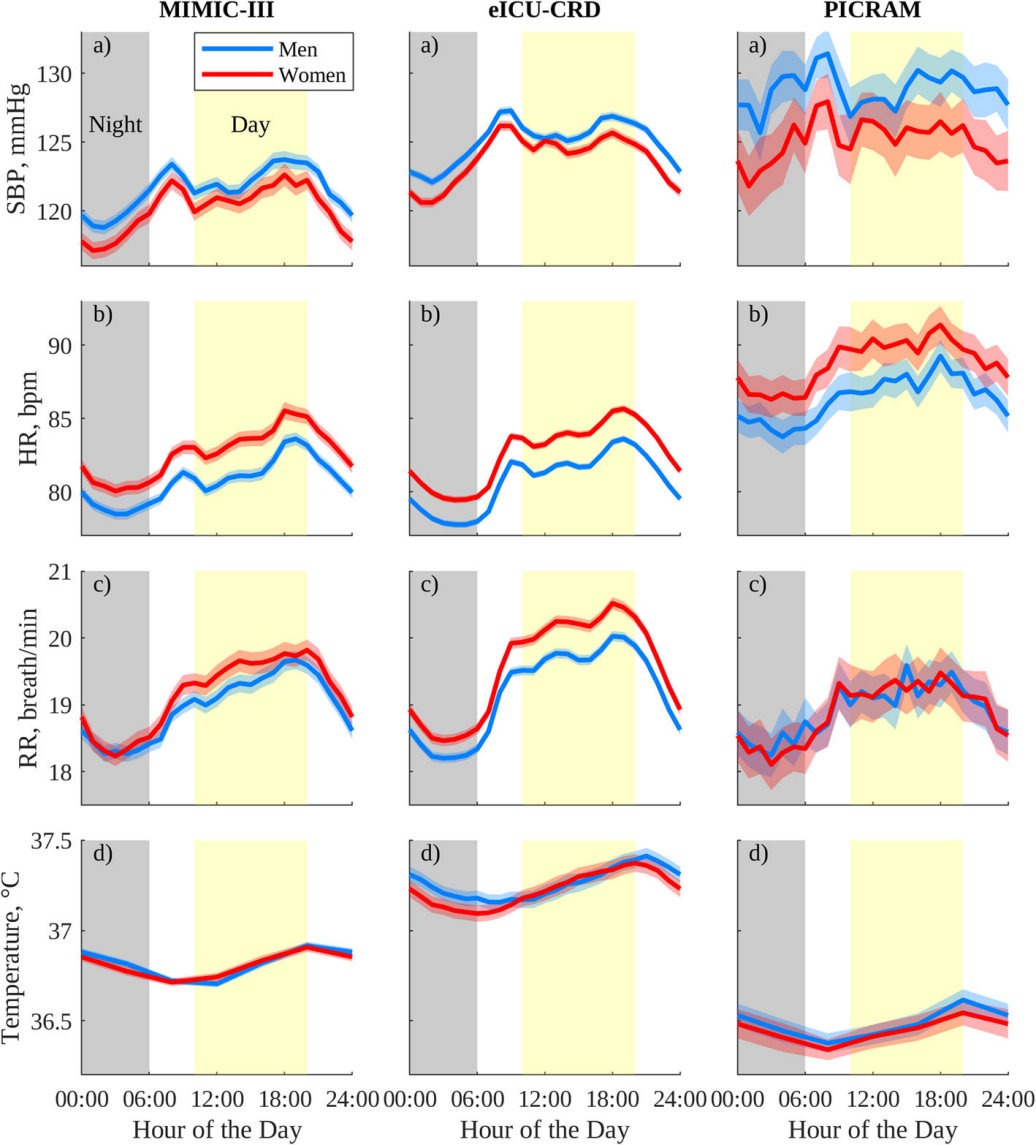
Circadian vital-sign profiles in the 24 h prior to discharge from the ICU for the selected patient cohorts for MIMIC-III, eICU-CRD, and PICRAM, grouped by gender: (a) SBP, (b) HR, (c) RR, and (d) T. The solid line represents the mean profile, and the shaded area the 95% CI of the population mean

Figure 1 shows the circadian profiles for SBP, HR, RR, and T grouped by gender for MIMIC-III, eICU-CRD, and PICRAM, with ‘night-time’ represented from 12 midnight–5:59 am and ‘day-time’ from 10 am–7:59 pm. By visual inspection, these profiles correspond well to those reported in healthy cohorts [20, 22] described previously and to those reported for non-ICU patients [34, 39]. In MIMIC-III and eICU-CRD, SBP is elevated between ≈ 2 and 14 h after the end of night-time, with three periodic maxima, though these are more pronounced than those observed in [20]. This elevated period is followed by a sinusoidal dip during night-time. HR in MIMIC-III and eICU-CRD is similarly elevated between ≈ 2 and 14 h after the end of night-time, again with three periodic maxima. Elevated SBP and reduced HR for men relative to women (*p* < 0.05, Fig. 1) also correspond to observations in [20]. In both SBP and HR, the smaller sample size in PICRAM results in more variability and makes features (especially maxima) more difficult to distinguish. However, the overall periods of elevated and reduced SBP and HR in PICRAM appear similar to those observed in MIMIC-III and eICU-CRD. The PICRAM cohort has an elevated mean HR (*p* < 0.05) relative to MIMIC-III and eICU-CRD and SBP (*p* < 0.05) relative to MIMIC-III.

There is a resemblance between the profiles in RR observed in Fig. 1 and the profiles in RR and V _E_ reported in [21] and [22], respectively. Elevated RR can be observed between ≈ 2 and 14 h after the end of night-time, peaking at 8 pm, with a trough between ≈ 2 and 6 am. T shows the expected sinusoidal behaviour [22, 40], though the low measurement frequency in MIMIC-III and PICRAM makes this harder to discern. T also shows a lag in the nadir of approximately 6–8 h relative to RR (and indeed SBP and HR) as observed relative to V _E_ in [22].

**Fig. 2:**
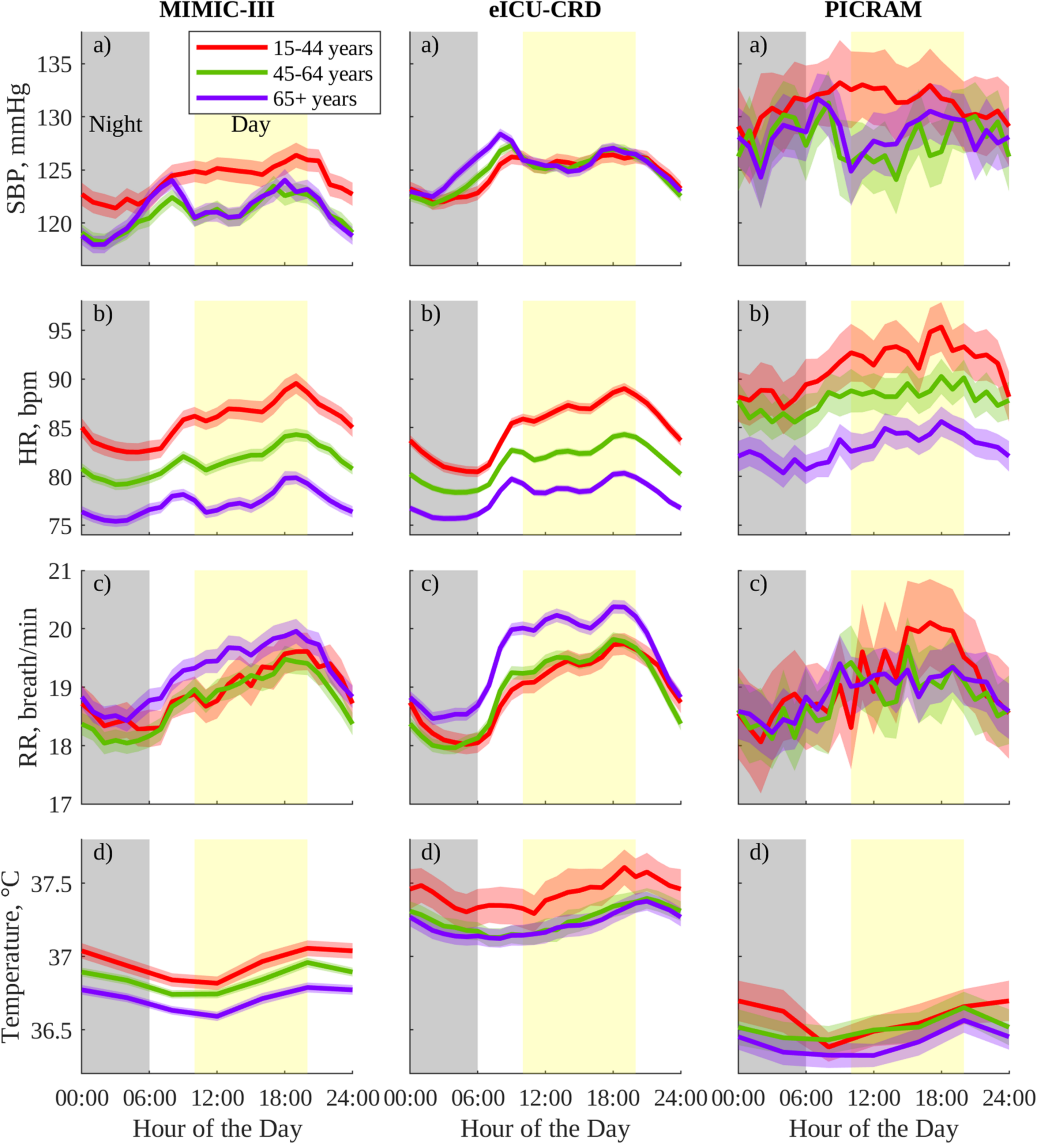
Circadian vital-sign profiles in the 24 h prior to discharge from the ICU for men in MIMIC-III, eICU-CRD, and PICRAM, grouped by age: (a) SBP, (b) HR, (c) RR, and (d) T. The solid line represents the mean profile, and the shaded area the 95% CI of the population mean for a group

**Fig. 3:**
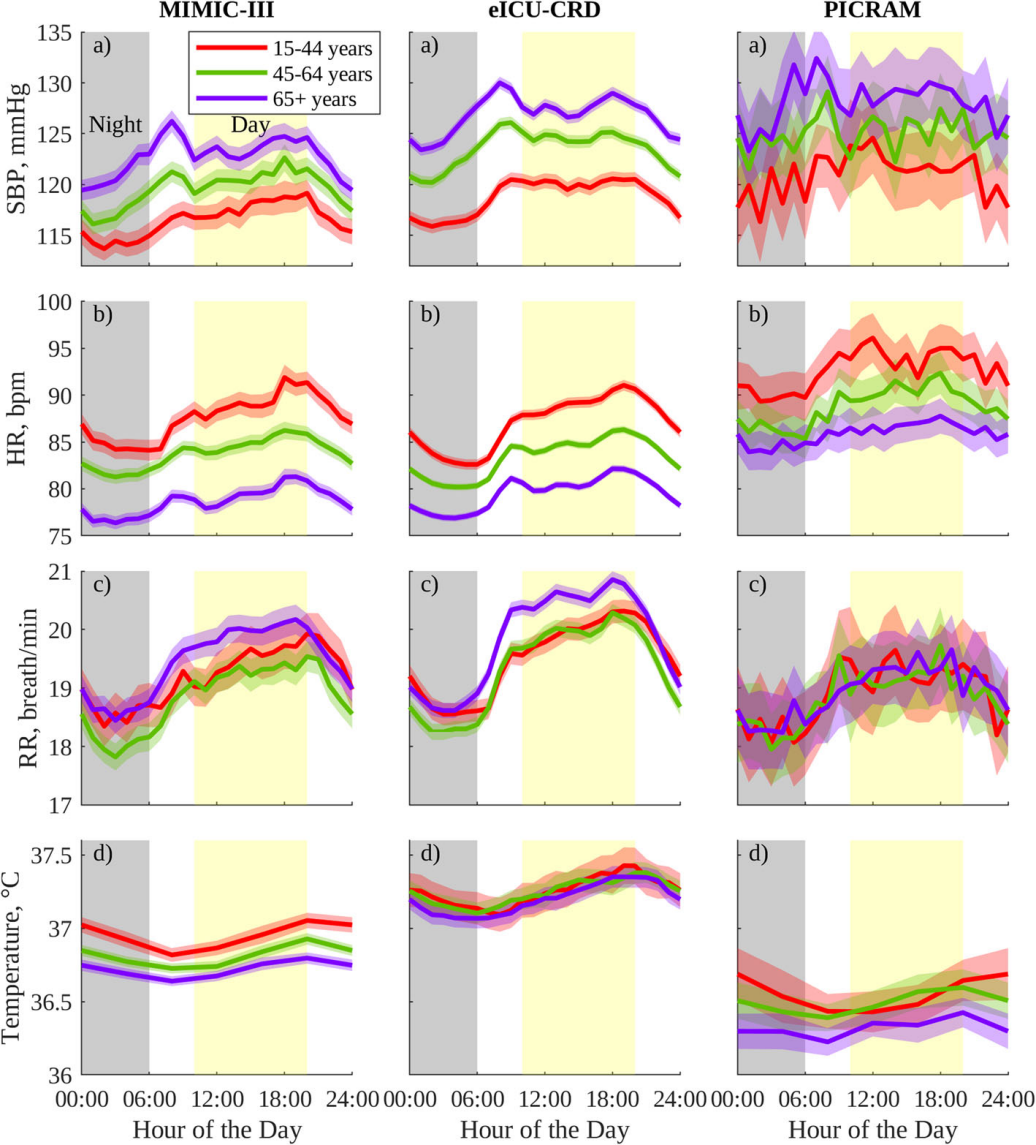
Circadian vital-sign profiles in the 24 h prior to discharge from the ICU for women in MIMIC-III, eICU-CRD, and PICRAM, grouped by age: (a) SBP, (b) HR, (c) RR, and (d) T. The solid line represents the mean profile, and the shaded area the 95% CI of the population mean for a group

The vital-sign patterns mentioned above largely hold for smaller cohorts grouped by gender and age, as shown in Fig. 2 for men and Fig. 3 for women. Once again, the smaller of these cohorts, such as those from PICRAM or the younger 15–44-year cohorts, show a greater degree of variability which makes features more difficult to distinguish. Figures 2 and 3 also show expected age-related trends [34]. These trends include progressively decreased HR in older age groups (*p* < 0.05) for MIMIC-III and eICU-CRD and between the 45–64 and 65+ groups in men in PICRAM. Women also show the expected increase in SBP with age (*p* < 0.05 for MIMIC-III and eICU-CRD); however, this trend is largely absent in men. A consistent increase in magnitude and duration of ascent prior to the morning (8:00 am) SBP peak across men and women as they age can be observed, similar to the trends reported in [34]. RR and T do not show clear variations with age, but both show consistent 24-h patterns across all age groups.

### Circadian vital-sign quantitative analyses

Table 4 shows that the peak-nadir excursions in all three ICU databases were attenuated relative to the peak-nadir excursions reported for non-ICU cohorts in the literature. The peak-nadir excursions for SBP and HR in the ICU cohorts in Table 4 are a factor of 4–5 times smaller than the values reported in [20]. Similarly, the peak-nadir excursions for RR in the ICU cohorts are a factor of 2 smaller than the value reported in [21], and the peak-nadir excursion in temperature is a factor of 2–3 times smaller than the corresponding value in [22].

**Table 4:** Peak-nadir excursion, expressed as raw value and as percentage of 24-h mean for vital signs. Overall data grouped by gender

	MIMIC-III		eICU-CRD		PICRAM		Literature^*^	
Vital sign	Men	Women	Men	Women	Men	Women	Men	Women
SBP, mmHg (%)	4.9 (4.1)	5.5 (4.6)	5.2 (4.2)	5.6 (4.5)	5.7 (4.5)	6.1 (4.9)	25.9 (22.3)	22.5 (21.1)
HR, bpm (%)	5.1 (6.4)	5.5 (6.6)	5.9 (7.2)	6.2 (7.5)	5.5 (6.4)	5.1 (5.7)	21.2 (30.5)	20.1 (25.9)
RR, breaths/min (%)	1.4 (7.4)	1.6 (8.3)	1.8 (9.5)	2.1 (10.5)	1.3 (7.1)	1.4 (7.3)	3.2 (16.8)	–
T, ^∘^C (%)	0.2 (0.6)	0.2 (0.5)	0.2 (0.6)	0.3 (0.7)	0.2 (0.7)	0.2 (0.6)	0.6 (1.6)	–

SBP and HR values from [20], RR values from [21], and temperature values from [22]

Table 5 shows that there is a strong correlation in vital-sign trends between all of the three databases. All vital-sign profiles were correlated between databases at the *p* = 0.05 level, and 20 of 24 correlated at the *p* = 0.01 level. Of the four exceptions, three were temperature profiles, where the lower *p* values observed were likely due to the lower frequency of the available measurements (once every 4 h).

**Table 5:** Pearson’s correlation coefficients (*R*) and *p* values (*p*) for inter-database vital-sign circadian pattern correlation. Data grouped by gender

	MIMIC-III and eICU-CRD		MIMIC-III and PICRAM		eICU-CRD and PICRAM	
Vital sign	Men	Women	Men	Women	Men	Women
SBP, *R* (*p*)	0.95 (0.00)	0.95 (0.00)	0.60 (0.00)	0.81 (0.00)	0.50 (0.01)	0.79 (0.00)
HR, *R* (*p*)	0.95 (0.00)	0.98 (0.00)	0.90 (0.00)	0.90 (0.00)	0.96 (0.00)	0.93 (0.00)
RR, *R* (*p*)	0.97 (0.00)	0.98 (0.00)	0.88 (0.00)	0.95 (0.00)	0.91 (0.00)	0.97 (0.00)
T, *R* (*p*)	0.88 (0.02)	0.84 (0.04)	0.92 (0.01)	0.97 (0.00)	0.98 (0.00)	0.88 (0.02)

## Discussion

### Presence of circadian rhythms

From Table 5, we can reasonably assert we are observing a generalisable vital-sign circadian ‘rhythm’ (i.e. a recurring vital-sign pattern with 24-h periodicity). This assertion is based on the high cross-correlations between 24-h vital-sign profiles from different databases, which are subject to different demographics and standards of care. That each individual’s contributing vital-sign profile may begin and end at any point within the 24 h adds further credence to the physiological, rather than environmental, origin of the observed rhythmicity.

Further evidence that we are observing vital-sign circadian rhythms is provided in Figs. 1, 2, and 3, where the observed vital-sign profiles show similar patterns across databases and with respect to those reported in the literature for non-ICU cohorts [20, 22, 40, 41]. Additionally, the relative trends between genders and between age groups are consistent with the literature, and across databases [34]. While a previous study [42] noted variations related to time of day in the agreement between nurse-verified and waveform-derived vital-sign measurements in MIMIC-II, these variations were of a ‘clinically insignificant amount’, and only measurement variability, not measurement bias, showed significant variation with time of day. As such, this behaviour is unlikely to contribute significantly to the observed profiles.

Overall, these results suggest observation of an intrinsic, consistent, demographically modified 24-h pattern in vital signs observable in the last 24 h prior to discharge from an ICU in the selected cohort of patients. This behaviour, observable across 50,298 ICU stays drawn from 211 hospitals across the UK and the USA with different patient demographics and standards of care, suggests that there is a typical circadian pattern in vital signs present in patients near recovery and discharge from an ICU.

### Rhythm topography

The peak-nadir excursions in SBP, HR, RR, and T (Table 4) were found to be 2–5 times smaller than those reported in the literature for healthy cohorts [20, 22]. There are several potential causes for this apparent attenuation of circadian amplitude. The suppression may be due to pathology or medication in the selected ICU cohort. The computation of average rhythms does not distinguish between amplitude attenuation caused by a mix of ‘healthy’ and attenuated rhythms and a consistent, cohort-wide attenuation, though the narrow 95% CIs of the mean would lend support to the latter. Alternatively, the observed reduced amplitudes may be demographically driven, as the results in both [20] and [22] are for young, healthy adults, and the results in [21] for competitive scuba divers, as opposed to the more heterogeneous, and generally older, cohort employed in this study. Also of note is that the data in both [20] and [22] are synchronised for waking time, rather than clock time, which may accentuate circadian rhythms. However, one would expect some degree of synchronicity in waking time within a given ICU, and for reduced synchronicity to ‘smear’ or laterally shift patterns rather than significantly decrease their peak amplitude.

A final potential cause of circadian amplitude attenuation is the fact that patients in an ICU are typically recumbent and physically inactive, which can affect circadian rhythm amplitude [43]. This consideration is supported by the fact that rhythm amplitude showed a factor of 4–5 times reduction in HR and SBP compared to [20], where subjects were ambulatory, but only a reduction of 2–3 times in RR and T compared to [21, 22], where patients were recumbent and inactive. Despite the intuitive appeal of these results, caution should be taken as [20–22] report different sets of vital signs using different protocols and equipment, and [21, 22] contain data from ≤ 10 individuals, making comparison difficult. Overall, it seems likely that amplitudes of circadian variation in vital signs are attenuated by some combination of pathology, treatment, and inactivity, with each vital sign responding differently.

### Variability between demographic cohorts

As previously mentioned, Figs. 2 and 3 largely show the expected age-related increase in mean SBP and decrease in mean HR [34, 44]. That these results are consistent across databases, and with results reported for non-ICU cohorts in the literature provides further support to the notion that the behaviour being observed is physiological behaviour, rather than behaviour governed by environmental influences.

However, mean SBP in men does not show age-related variations despite these being well documented in healthy men and present for women in the selected cohort [34]. It is important to note that the ICU population for a given demographic group is not necessarily representative of the general population for that demographic group, and this is elaborated further in Additional file 3. Broadly, young men (between 15 and 44 years) have a relatively high prevalence of admission diagnoses codes for HIV, alcohol abuse, and trauma not seen in younger or older women, or in older men. These variations in cause of ICU admission, and thus patient condition and treatment, may explain this lack of expected trends with age in mean SBP for men.

### Variability between databases

As previously mentioned, PICRAM shows both an increased retention rate in the selected cohort (Table 3) and an elevated mean HR and SBP (Fig. 1). It is likely the increased retention rate of PICRAM ICU stays relative to MIMIC-III or eICU-CRD is due in part to the lack of discharge destination coding in the UK, leading to all patients expected to make a full recovery in PICRAM being retained, as opposed to only those discharged home as in MIMIC-III and eICU-CRD. Thus, it is likely that the increase in mean HR and SBP observed in PICRAM is due to the PICRAM cohort being older and more ill, rather than local variables or changes in clinical practice. These observations correspond to data present in the literature that suggest that patients in UK ICUs are on average more ill than those in US ICUs, associated with the lower number of ICU beds per capita available in the UK [45, 46]. It is important to note that the circadian pattern shapes and intra-database trends with gender and age hold across all three databases, regardless of differences in clinical behaviour or shift timings, suggesting these profiles are widely generalisable.

### Limitations

This study has several limitations that are worth discussing. All trends reported in this paper are for the average of large numbers of vital-sign measurements across a reasonably diverse cohort of patients. Thus, while the trends observed match trends reported for healthy individuals outside the ICU, and the trends are generally maintained when the data are broken up into subgroups by gender or age, there is little indication as to how consistently these trends can be observed on an individual basis. This is important as any prospective tracking of patient recovery, or of the development of complications such as delirium [5], would require the ability to meaningfully establish an individual’s vital-sign circadian rhythms using routine clinical measurements.

While a large amount of patient data has been gathered across a large number of different hospitals, it also worth noting that data are still only gathered from 2 countries with lifestyles and demographics that are reasonably similar. Thus, further work is required to assess the generalisability of any trends observed to other countries where diet, clinical practice, and cause of ICU admission may vary to a greater extent.

This paper does not compare circadian rhythmicity between patients who ‘recovered’ and those who died. As such, the paper does not provide evidence of the ‘sensitivity’ of observable circadian vital-sign patterns to patient recovery, only that this behaviour can be observed in those who recovered. Research into generalisable circadian vital-sign behaviour in the ICU is relatively new. As such, it is important to establish that generalisable circadian behaviour exists prior to discharge in ICU patients who recovered, thus laying the groundwork for future comparisons between cohorts.

This paper does not demonstrate loss of circadian rhythmicity in the selected cohorts earlier in their ICU stay. Instead, it relies on existing literature that suggests circadian rhythms are severely disrupted in an ICU [3, 4, 15, 23]. Patients early in an ICU stay are likely to have their vital-sign patterns directly disrupted by medication and clinical interventions, making observation of any underlying circadian pattern, whether present or not, significantly more challenging. Finally, this paper seeks to evaluate circadian rhythmicity in the last 24 h prior to discharge from an ICU in the typical ICU patient who recovered. As such, the relatively short stay of ICU patients in the US databases, attributable to broader intake criteria used in US ICUs [45, 46], should be noted.

## Conclusion

This paper investigated the presence of, and the relationships between, circadian rhythms in SBP, HR, RR, and T across a subset of patients in the MIMIC-III, eICU-CRD, and PICRAM ICU databases deemed most likely to exhibit circadian behaviour. Circadian patterns in SBP, HR, RR, and T that visually corresponded to those reported in the literature for non-ICU cohorts were observed, and these circadian patterns showed strong correlations between databases (mean *R* of 0.89). The peak-nadir excursions of the observed circadian patterns were reduced by a factor of 2–5 compared to behaviour reported in the literature for young, healthy individuals. These results support the existence of circadian rhythms in ICU patients who are within 24 h of discharge, and the generalisability of these circadian patterns across different cohorts subject to different standards of clinical practice. The existence of a generalisable circadian state prior to ICU discharge in patients who recovered has potential application in both prospective and retrospective tracking of patient recovery in the ICU, as well as the development of complications such as delirium.

## Supplementary information

**Additional file 1** Data selection. Description of data: This PDF contains details of the criteria used to select patients and vital sign measurements for this study, as well as tables of the number of patients, ICU stays, and vital sign measurements that met each criterion.

**Additional file 2** Number of measurements. Description of data: This PDF contains details of the number of measurements in the selected cohort available for a given database and vital sign at any given hour.

**Additional file 3** Admission diagnoses. Description of data: This PDF contains details of admission diagnoses of the selected cohort of patients from each database, with commentary on potential reasons for the trend with age in mean SBP in young men (aged 15 - 44 years) does not correspond to that reported in non-ICU cohorts in literature.

## Data Availability

The datasets analysed during the current study are available in the following repositories: MIMIC-III (https://mimic.physionet.org/), eICU-CRD (https://eicu-crd.mit.edu/), and PICRAM (http://www.isrctn.com/ISRCTN32008295).

## Abbreviations

ICU: Intensive care unit
EEG: Electroencephalography
SBP: Systolic blood pressure
HR: Heart rate
RR: Respiratory rate
T: Temperature
MIMIC-III: Medical Information Mart for Intensive Care III
eICU-CRD: eICU Collaborative Research Database
PICRAM: Post-Intensive-Care Risk-adjusted Alerting and Monitoring
BIDMC: Beth Israel Deaconess Medical Centre
HIPAA: Health Insurance Portability and Accountability Act
CI: Confidence interval
OASIS: Oxford Acute Severity of Illness Score

## References

[1] Gotts JE, Matthay MA (2016). Sepsis: pathophysiology and clinical management. Bmj.

[2] Holst LB, Haase N, Wetterslev J, Wernerman J, Guttormsen AB, Karlsson S (2014). Lower versus higher hemoglobin threshold for transfusion in septic shock. N Engl J Med.

[3] McKenna H, van der Horst, Reiss I, Martin D (2018). Clinical chronobiology: a timely consideration in critical care medicine. Critical Care.

[4] Papaioannou V, Mebazaa A, Plaud B, Legrand M (2014). ’Chronomics’ in ICU: circadian aspects of immune response and therapeutic perspectives in the critically ill. Intensive Care Med Exp.

[5] Durrington HJ (2017). Light intensity on intensive care units-a short review. J Intensive Crit Care.

[6] Lamia KA. Ticking time bombs: connections between circadian clocks and cancer. F1000Research. 2017; 6. 10.12688/f1000research.11770.1. 10.12688/f1000research.11770.1 PMC5664980 29152229

[7] Young ME, Bray MS (2007). Potential role for peripheral circadian clock dyssynchrony in the pathogenesis of cardiovascular dysfunction. Sleep Med.

[8] Roenneberg T, Allebrandt KV, Merrow M, Vetter C (2012). Social jetlag and. Curr Biol.

[9] Wong PM, Hasler BP, Kamarck TW, Muldoon MF, Manuck SB (2015). Social jetlag, chronotype, and cardiometabolic risk. J Clin Endocrinol Metabol.

[10] Born J, Lange T, Hansen K, Mölle M, Fehm HL (1997). Effects of sleep and circadian rhythm on human circulating immune cells. J Immunol.

[11] Estrup S, Kjer C, Poulsen L, Gogenur I, Mathiesen O (2018). Delirium and effect of circadian light in the intensive care unit: a retrospective cohort study. Acta Anaesthesiol Scand.

[12] Freedman NS, Gazendam J, Levan L, Pack AI, Schwab RJ (2001). Abnormal sleep/wake cycles and the effect of environmental noise on sleep disruption in the intensive care unit. Am J Respir Crit Care Med.

[13] Ely EW, Shintani A, Truman B, Speroff T, Gordon SM, Harrell Jr FE (2004). Delirium as a predictor of mortality in mechanically ventilated patients in the intensive care unit. Jama.

[14] Li J, Li R, Gao Y, Zhang J, Zhao Y, Zhang X (2019). Nocturnal mean arterial pressure rising is associated with mortality in the intensive care unit: a retrospective cohort study. J Am Heart Assoc.

[15] Telias I, Wilcox ME (2019). Sleep and circadian rhythm in critical illness. Crit Care.

[16] Silber MH, Ancoli-Israel S, Bonnet MH, Chokroverty S, Grigg-Damberger MM, Hirshkowitz M (2007). The visual scoring of sleep in adults. J Clin Sleep Med.

[17] Boyko Y, Jennum P, Toft P (2017). Sleep quality and circadian rhythm disruption in the intensive care unit: a review. Nat Sci Sleep.

[18] Cooper AB, Thornley KS, Young GB, Slutsky AS, Stewart TE, Hanly PJ (2000). Sleep in critically ill patients requiring mechanical ventilation. Chest.

[19] Watson PL, Pandharipande P, Gehlbach BK, Thompson JL, Shintani AK, Dittus BS, et al.Atypical sleep in ventilated patients: empirical electroencephalography findings and the path toward revised ICU sleep scoring criteria. Crit Care Med. 2013; 41(8). 10.1097/CCM.0b013e31828a3f75 PMC3875326 23863228

[20] Hermida RC, Ayala DE, Fernández JR, Mojón A, Alonso I, Calvo C (2002). Modeling the circadian variability of ambulatorily monitored blood pressure by multiple-component analysis. Chronobiol Int.

[21] Bosco G, Ionadi A, Panico S, Faralli F, Gagliardi R, Data P (2003). Effects of hypoxia on the circadian patterns in men. High Altitude Med Biol.

[22] Spengler CM, Czeisler CA, Shea SA (2000). An endogenous circadian rhythm of respiratory control in humans. J Physiol.

[23] Brito RA, Viana SMdNR, Beltrão BA, de Araújo Magalhães CB, de Bruin VMS, de Bruin PFC. Pharmacological and non-pharmacological interventions to promote sleep in intensive care units: a critical review. Sleep Breathing. 2019:1–11. 10.1007/s11325-019-01902-7. 10.1007/s11325-019-01902-7 31368029

[24] Johnson AE, Pollard TJ, Shen L, Li-wei HL, Feng M, Ghassemi M (2016). MIMIC-III, a freely accessible critical care database. Sci Data.

[25] Saeed M, Villarroel M, Reisner AT, Clifford G, Lehman LW, Moody G (2011). Multiparameter Intelligent Monitoring in Intensive Care II (MIMIC-II): a public-access intensive care unit database. Crit Care Med.

[26] Pollard TJ, Johnson AE, Raffa JD, Celi LA, Mark RG, Badawi O. The eICU Collaborative Research Database, a freely available multi-center database for critical care research. Sci Data. 2018; 5. 10.1038/sdata.2018.178. 10.1038/sdata.2018.178 PMC6132188 30204154

[27] Berry W, McKenzie C (2010). Use of inotropes in critical care. Clin Pharm.

[28] McEvoy GK. AHFS drug information, 2000: American Society of Health-System Pharmacists; 2000.

[29] Babar SM (2013). SIADH associated with ciprofloxacin. Ann Pharm.

[30] White PF (1988). Propofol: pharmacokinetics and pharmacodynamics. Semin Anesth.

[31] Idama TO, Lindow SW (1998). Magnesium sulphate: a review of clinical pharmacology applied to obstetrics. BJOG: Int J Obstet Gynaecol.

[32] Regårdh CG, Borg KO, Johansson R, Johnsson G, Palmer L (1974). Pharmacokinetic studies on the selective *β* 1-receptor antagonist metoprolol in man. J Pharmacokinet Biopharm.

[33] O’Malley K, Segal J, Israili Z, Boles M, McNay J, Dayton P (1975). Duration of hydralazine action in hypertension. Clin Pharm Ther.

[34] Mahdi A, Watkinson P, McManus RJ, Tarassenko L (2019). Circadian blood pressure variations computed from 1.7 million measurements in an acute hospital setting. Am J Hypertens.

[35] Department of International Economic and Social Affairs (1982). Provisional Guidelines on Standard International Age Classifications.

[36] Johnson AE, Kramer AA, Clifford GD (2013). A new severity of illness scale using a subset of acute physiology and chronic health evaluation data elements shows comparable predictive accuracy. Crit Care Med.

[37] Cousins L, Rigg L, Hollingsworth D, Meis P, Halberg F, Brink G (1983). Qualitative and quantitative assessment of the circadian rhythm of cortisol in pregnancy. Am J Obstet Gynecol.

[38] Nagele P (2003). Misuse of standard error of the mean (SEM) when reporting variability of a sample. A critical evaluation of four anaesthesia journals. Br J Anaesth.

[39] Ceyhan M, Günaydin S, Yorgancioglu C, Zorlutuna Y, Uluoglu C, Zengil H (2003). Comparison of circadian rhythm characteristics of blood pressure and heart rate in patients before and after elective coronary artery bypass surgery. Chronobiol Int.

[40] Vitiello MV, Smallwood RG, Avery DH, Pascualy RA, Martin DC, Prinz PN (1986). Circadian temperature rhythms in young adult and aged men. Neurobiol Aging.

[41] Malpas SC, Purdie GL (1990). Circadian variation of heart rate variability. Cardiovasc Res.

[42] Hug C, Clifford GD. An analysis of the errors in recorded heart rate and blood pressure in the ICU using a complex set of signal quality metrics. In: 2007 Computers in. IEEE: 2007. p. 641–4. 10.1109/cic.2007.4745567.

[43] Mortola JP (2004). Breathing around the clock: an overview of the circadian pattern of respiration. Eur J Appl Physiol.

[44] Bonnemeier H, Wiegand UK, Brandes A, Kluge N, Katus HA, Richardt G (2003). Circadian profile of cardiac autonomic nervous modulation in healthy subjects: differing effects of aging and gender on heart rate variability. J Cardiovasc Electrophysiol.

[45] Prin M, Wunsch H (2012). International comparisons of intensive care: informing outcomes and improving standards. Curr Opin Crit Care.

[46] Wunsch H, Angus DC, Harrison DA, Linde-Zwirble W T Rowan (2011). Comparison of medical admissions to intensive care units in the United States and United Kingdom. Am J Respir Crit Care Med.

