# Correction to: Vital-sign circadian rhythms in patients prior to discharge from an ICU: a retrospective observational analysis of routinely recorded physiological data

**DOI:** 10.1186/s13054-022-03887-4

**Published:** 2022-01-17

**Authors:** Shaun Davidson, Mauricio Villarroel, Mirae Harford, Eoin Finnegan, Joao Jorge, Duncan Young, Peter Watkinson, Lionel Tarassenko

**Affiliations:** 1 Institute of Biomedical Engineering, Department of Engineering Science, University of Oxford, Oxford, UK; 2 Critical Care Research Group, Nuffield Department of Clinical Neurosciences, University of Oxford, Oxford, UK

## Correction to: Critical Care (2020) 24:181 10.1186/s13054-020-02861-2

Following publication of the original article [1], the authors identified an error in Fig. 2. The error was that Figs. 2 and 3 were both displaying the same figure. The correct Fig. 2 is given hereafter.

**Fig. 2:**
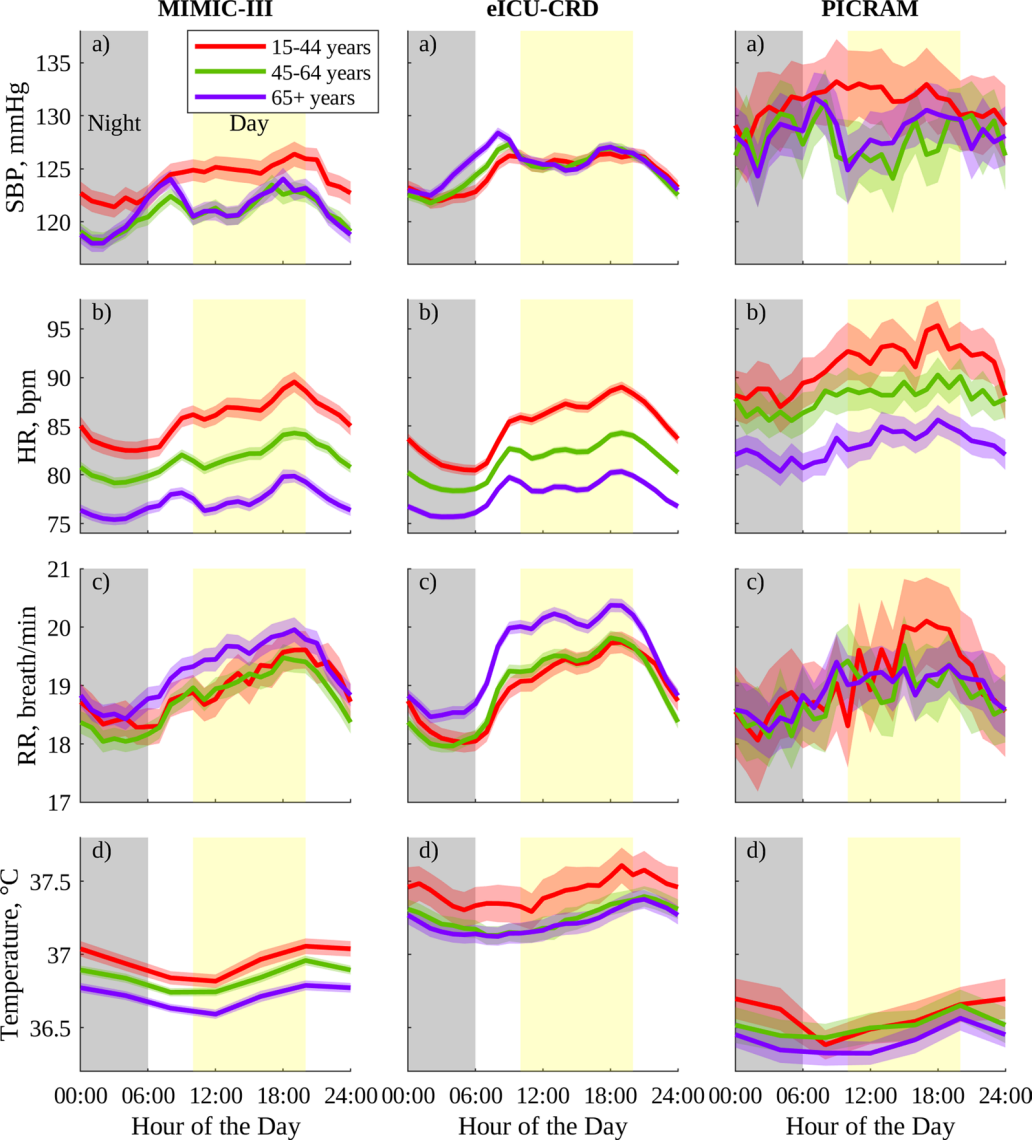
Circadian vital-sign profiles in the 24 h prior to discharge from the ICU for men in MIMIC-III, eICU-CRD, and PICRAM, grouped by age: **a** SBP, **b** HR, **c** RR, and **d** T. The solid line represents the mean profile, and the shaded area the 95% CI of the population mean for a group

All the changes that were requested are implemented in this correction and the original article [1] has been corrected.
