# Supplementary material for: Vital-sign circadian rhythms in patients prior to discharge from an ICU: a retrospective observational analysis of routinely recorded physiological data
Source: Crit Care. 2020 Apr 28;24:181. doi: 10.1186/s13054-020-02861-2 (PMC7189546; doi:10.1186/s13054-020-02861-2)
Supplement: Supplementary file 1 — Additional file 1 Data selection. Description of data: This PDF contains details of the criteria used to select patients and vital sign measurements for this study, as well as tables of the number of patients, ICU stays, and vital sign measurements that met each criterion. [file 13054_2020_2861_MOESM1_ESM.pdf]

ADDITIONAL FILE 1

Vital-sign circadian rhythms in patients prior to discharge from an ICU: A retrospective observational analysis of routinely recorded physiological data

Shaun Davidson[1*], Mauricio Villarroel[1], Mirae Harford[1,2], Eoin Finnegan[1], Joao Jorge[1], Duncan Young[2], Peter Watkinson[2] and Lionel Tarassenko[1]

[*]Correspondence: shaun.davidson@eng.ox.ac.uk
[1]Institute of Biomedical Engineering, Department of Engineering Science, University of Oxford, Oxford, UK
Full list of author information is available at the end of the article

Medication Exclusion Criteria

Overall, the goal of the medication exclusion criteria was to exclude medication that met the following criteria:

- Would have a significant, rapid onset effect on patient vital signs, such that underlying trends in vital signs would be masked.
- Would potentially be given to a significant proportion of patients within the last 24 hours prior to discharge from an ICU.

Where possible, chronic, or longer term medication which was unlikely to significantly affect vital-sign trends hour-to-hour were not excluded. Additionally, administration of such chronic medication is often temporarily halted while patients are in an ICU and only restarted after discharge. Inclusion of patients on chronic medication was important in providing a reasonable picture of a recovering ICU patient, and retaining sufficient data for analysis. β-blockers, which are used orally as a treatment for chronic hypertension, were excluded when given intravenously, as this can have significant rapid onset effects on blood pressure and heart rate. The current exclusion criteria result in a reduction in vital-sign measurements of between 10.3% and 25.4% depending on the database (table 3 in the main text), thus the majority of patient data is retained.

Figs. 1 - 3 show the proportion of patients in the selected cohort who were treated by a broader list of vital sign altering medication within the last 26 hours (allowing for a ~2 hour duration of action) of an ICU stay. It can be seen that the non-excluded medications administered most frequently to patients during this period were typically medications with longer-term effects (e.g. dexmedetomidine - a sedative which, notably, does not cause respiratory effects, and diuretics, which act more indirectly on blood pressure). Fewer than 3% of patients in the selected cohort in each database are administered any given benzodiazepine or ACE inhibitor, and these medications are unlikely to dramatically alter vital signs in the short term. Calcium channel antagonists (e.g. Amlodipine, Diltiazem, Nicardepine) are more common, with 9.53% of PICRAM patients having Amlodipine and 8.68% of patients in eICU-CRD having Nicardipine in the final day.

Author details
[1]Institute of Biomedical Engineering, Department of Engineering Science, University of Oxford, Oxford, UK.
[2]Critical Care Research Group, Nuffield Department of Clinical Neurosciences, University of Oxford, Oxford, UK.

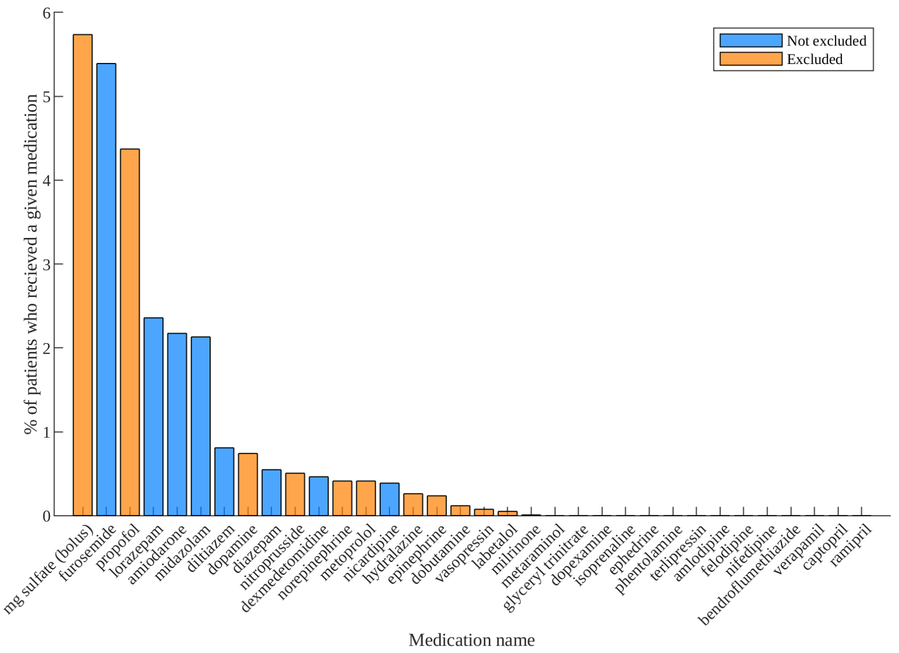

Figure 1 The proportion of patients in the selected cohort from MIMIC-III who received any of a broader range of vital sign altering medication within the final 26 hours of their ICU stay.

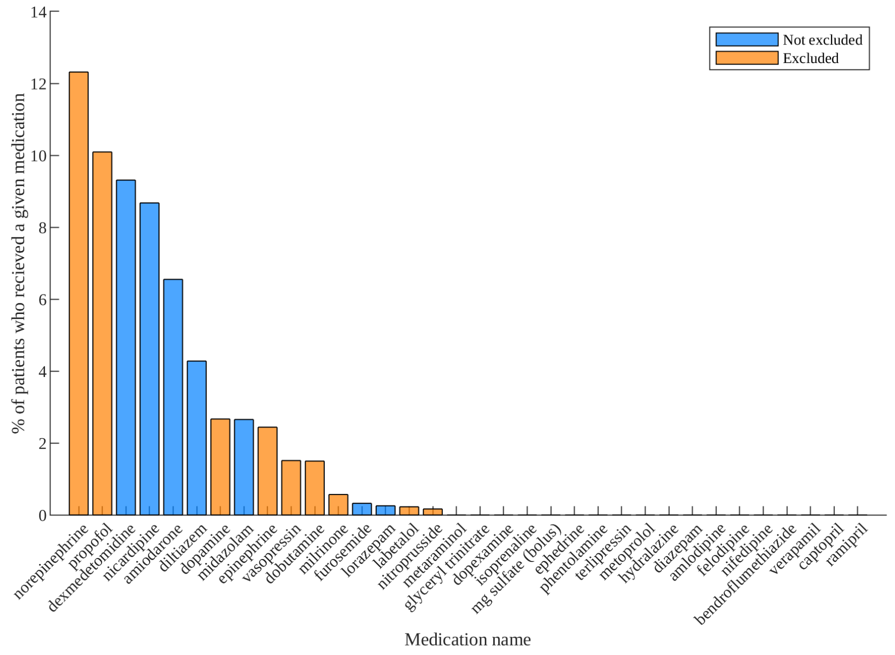

Figure 2 The proportion of patients in the selected cohort from eICU-CRD who received any of a broader range of vital sign altering medication within the final 26 hours of their ICU stay.

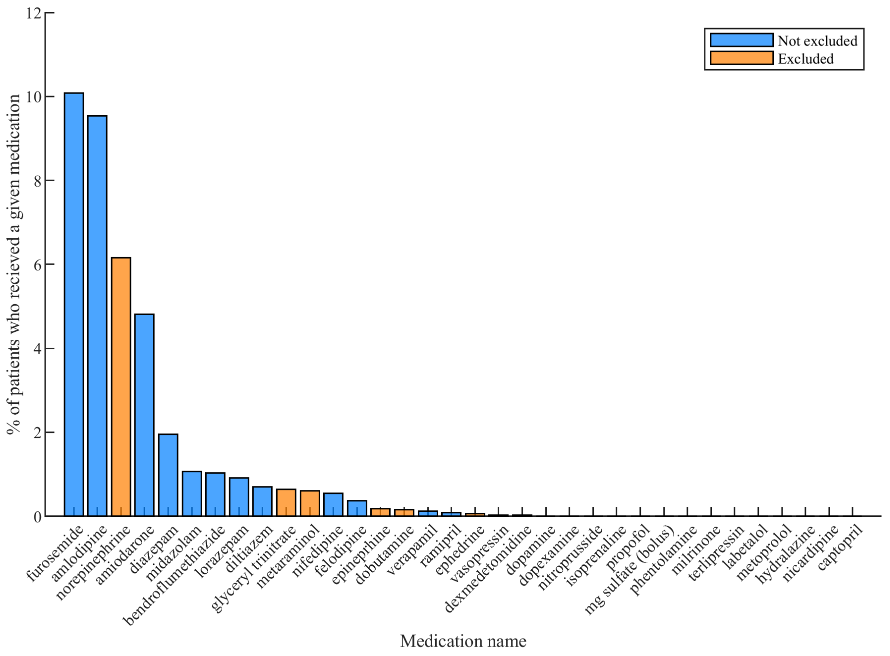

Figure 3 The proportion of patients in the selected cohort from PICRAM who received any of a broader range of vital sign altering medication within the final 26 hours of their ICU stay.

References
