# Supplementary material for: Vital-sign circadian rhythms in patients prior to discharge from an ICU: a retrospective observational analysis of routinely recorded physiological data
Source: Crit Care. 2020 Apr 28;24:181. doi: 10.1186/s13054-020-02861-2 (PMC7189546; doi:10.1186/s13054-020-02861-2)
Supplement: Supplementary file 2 — Additional file 2 Number of measurements. Description of data: This PDF contains details of the number of measurements in the selected cohort available for a given database and vital sign at any given hour. [file 13054_2020_2861_MOESM2_ESM.pdf]

ADDITIONAL FILE 2

Vital-sign circadian rhythms in patients prior to discharge from an ICU: A retrospective observational analysis of routinely recorded physiological data

Shaun Davidson[1*], Mauricio Villarroel[1], Mirae Harford[1,2], Eoin Finnegan[1], Joao Jorge[1], Duncan Young[2], Peter Watkinson[2] and Lionel Tarassenko[1]

[*]Correspondence: shaun.davidson@eng.ox.ac.uk
[1]Institute of Biomedical Engineering, Department of Engineering Science, University of Oxford, Oxford, UK
Full list of author information is available at the end of the article

Distribution of Measurements

This additional file shows the number of measurements averaged to create each of the 24-hourly data point plotted in the vital-sign profile figures. For all databases and vital signs, the number of measurements in each hourly bin that met the employed selection criteria was relatively consistent. MIMIC-III shows the clearest visible variations in frequency of measurements that met the selection criteria with time-of-day, with a maximum decrease in the number of such measurements by 32.0% - 37.7% depending on vital sign (from peak) in the late afternoon. As the majority of patients are weaned off vital sign altering medication near to discharge from the ICU, there is a higher proportion of patients on such medication, for example, 20 - 24 hours prior to discharge. As a large number of patients are discharged during the late afternoon, there is an increased likelihood of data being excluded from the late afternoon on the day before discharge. Interestingly, this reduction is not as clearly observable in the eICU-CRD data. Regardless, there is a significant and broadly similar amount of patient data available at any given time-of-day for each vital sign within each database.

Author details
[1]Institute of Biomedical Engineering, Department of Engineering Science, University of Oxford, Oxford, UK.
[2]Critical Care Research Group, Nuffield Department of Clinical Neurosciences, University of Oxford, Oxford, UK.

References

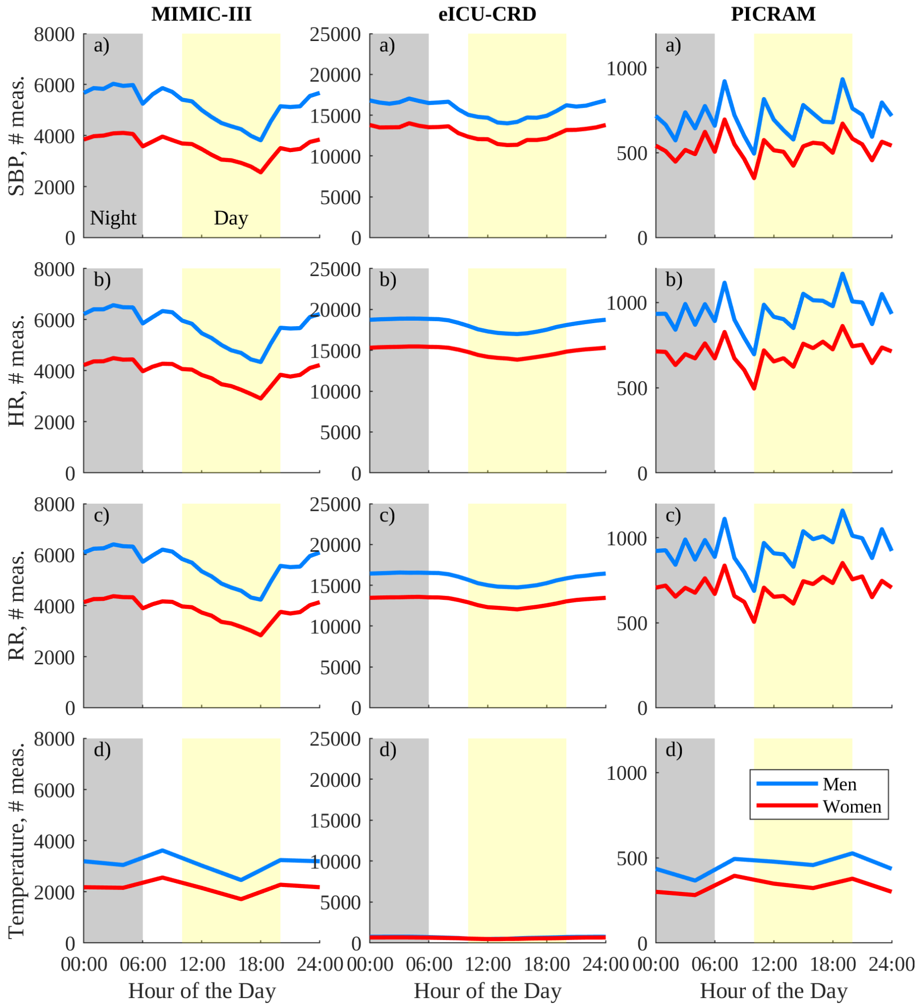

Figure 1 No. of measurements used to construct 24-hour circadian vital-sign profiles for the selected patient cohorts for MIMIC, eICU-CRD, and PICRAM, grouped by gender: a) SBP; b) HR; c) RR; d) T.
