# Supplementary material for: Vital-sign circadian rhythms in patients prior to discharge from an ICU: a retrospective observational analysis of routinely recorded physiological data
Source: Crit Care. 2020 Apr 28;24:181. doi: 10.1186/s13054-020-02861-2 (PMC7189546; doi:10.1186/s13054-020-02861-2)
Supplement: Supplementary file 3 — Additional file 3 Admission diagnoses. Description of data: This PDF contains details of admission diagnoses of the selected cohort of patients from each database, with commentary on potential reasons for the trend with age in mean SBP in young men (aged 15 - 44 years) does not correspond to that reported in non-ICU cohorts in literature. [file 13054_2020_2861_MOESM3_ESM.pdf]

ADDITIONAL FILE 3

Vital-sign circadian rhythms in patients prior to discharge from an ICU: A retrospective observational analysis of routinely recorded physiological data

Shaun Davidson[1*], Mauricio Villarroel[1], Mirae Harford[1,2], Eoin Finnegan[1], Joao Jorge[1], Duncan Young[2], Peter Watkinson[2] and Lionel Tarassenko[1]

[*]Correspondence: shaun.davidson@eng.ox.ac.uk
[1]Institute of Biomedical Engineering, Department of Engineering Science, University of Oxford, Oxford, UK
Full list of author information is available at the end of the article

Admission diagnoses

Figs. 1, 2 and 3 present the 15 most common diagnosis codes for different age and gender subgroups. Note that MIMIC-III and eICU-CRD used the ICD-9 coding system for admission diagnoses, while PICRAM used the ICNARC Coding Method (ICM). All analysis of diagnosis codes in this section is performed using up to the top 3 diagnosis codes for each patient. Note that the normalised frequency plotted is the proportion of ICU admissions, not admission diagnosis codes, that match a given admission diagnosis. Thus, for up to three diagnosis codes per patient, the total sum of normalised frequency will be between 1 and 3, rather than equal to 1.

From these figures, it is apparent that the younger 15 - 44 year cohorts of both genders have notably more varied causes of admission than the older 45 - 90+ year cohorts. Men in the younger cohort had the highest prevalence of diagnosis codes related to alcohol abuse, HIV, and trauma, by a considerable margin. Thus, younger men were one of the more heterogeneous cohorts, with a higher prevalence of HIV, alcohol, and trauma-related intake codes. Older men, in contrast, were a more homogeneous cohort. Women show a less dramatic variance in cohort heterogeneity with age, and younger women, while still heterogeneous, show a lower prevalence of alcohol-related, trauma-related, or otherwise unique admission diagnosis codes than younger men. This contrast may explain the lack of a clear upward trend in mean SBP with age in men, compared to the expected upward trend in mean SBP with age observed in women.

Author details
[1]Institute of Biomedical Engineering, Department of Engineering Science, University of Oxford, Oxford, UK.
[2]Critical Care Research Group, Nuffield Department of Clinical Neurosciences, University of Oxford, Oxford, UK.

References

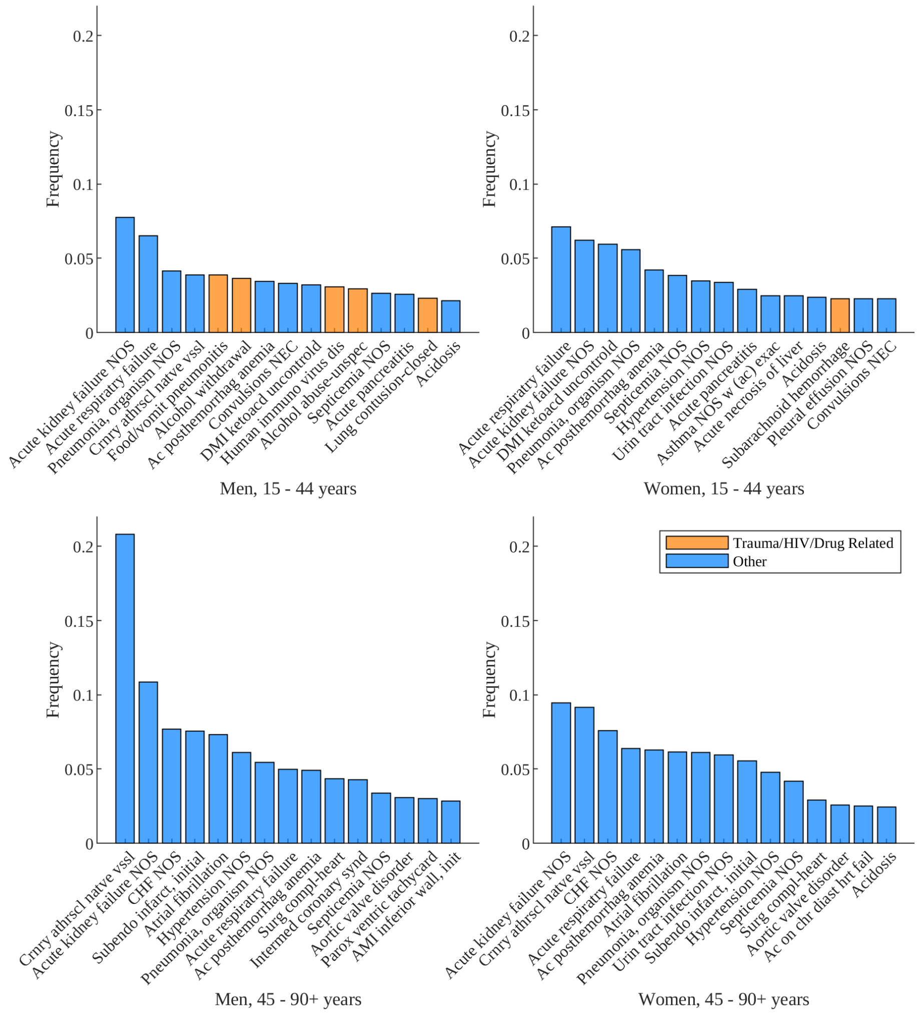

Figure 1 15 most common diagnosis codes for MIMIC patient cohorts grouped by gender and age. Orange columns diagnosis codes associated with HIV, trauma, or drug abuse. Abbreviations: NOS - Not Otherwise Specified, NEC - Not Elsewhere Classified, DMI - Diabetes Mellitus Type I, CHF - Coronary Heart Failure.

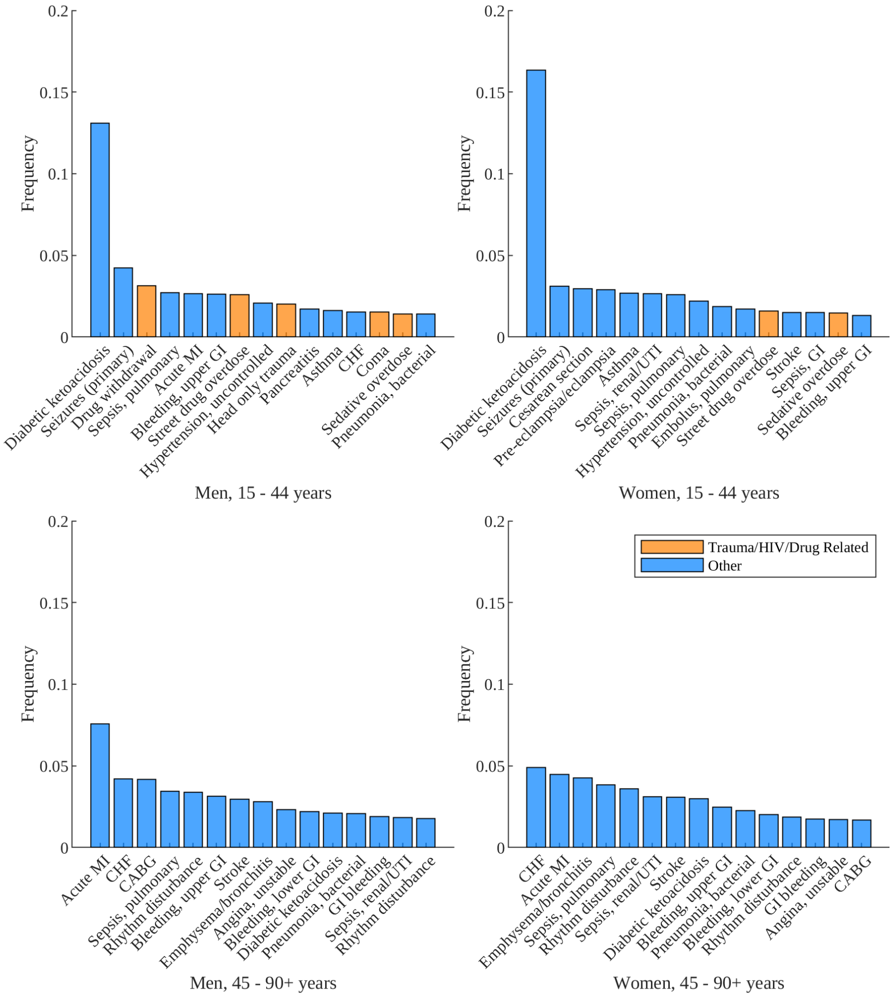

Figure 2 15 most common diagnosis codes for eICU-CRD patient cohorts grouped by gender and age. Orange columns diagnosis codes associated with HIV, trauma, or drug abuse. Abbreviations: MI - Myocardial Infarction, GI - Gastro-intestinal, CHF - Coronary Heart Failure, UTI - Urinary Tract Infection, CABG - Coronary Artery Bypass Grafting.

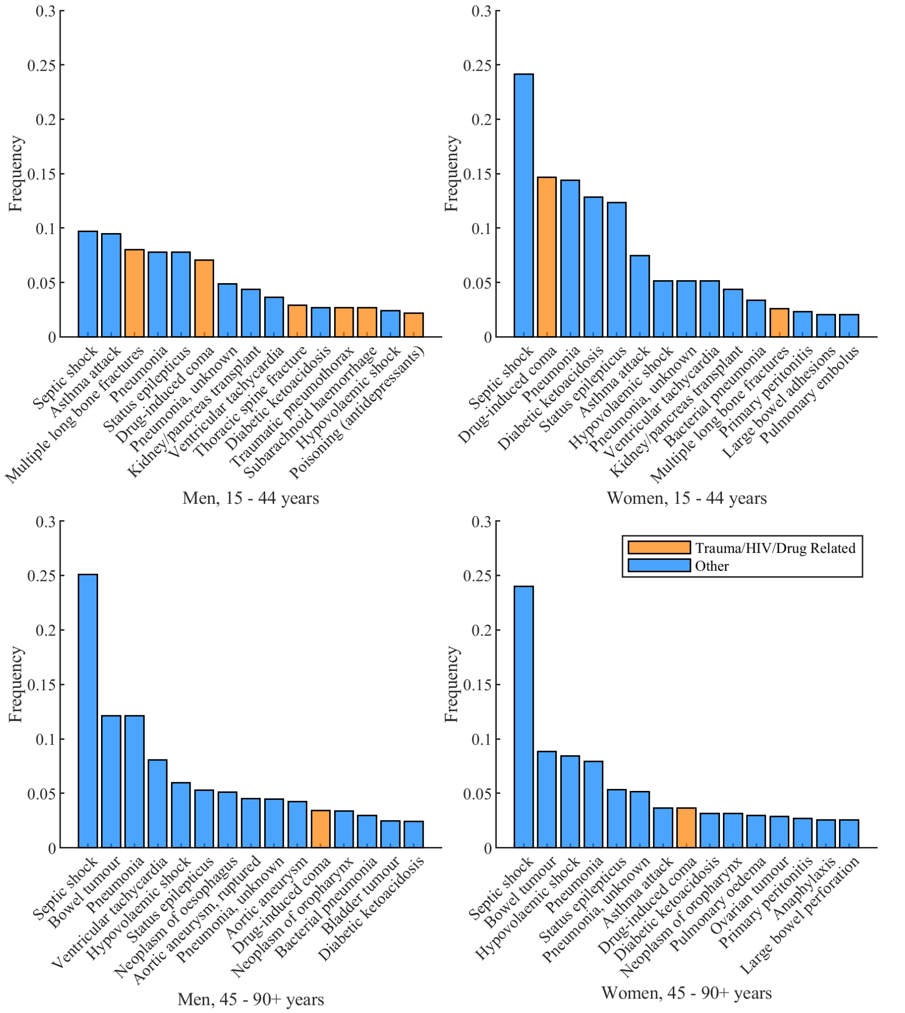

Figure 3 15 most common diagnosis codes for PICRAM patient cohorts grouped by gender and age. Orange columns diagnosis codes associated with HIV, trauma, or drug abuse.
